# How Blackouts during Heat Waves Amplify Mortality and Morbidity Risk

**DOI:** 10.1021/acs.est.2c09588

**Published:** 2023-05-23

**Authors:** Brian Stone, Carina J. Gronlund, Evan Mallen, David Hondula, Marie S. O’Neill, Mayuri Rajput, Santiago Grijalva, Kevin Lanza, Sharon Harlan, Larissa Larsen, Godfried Augenbroe, E. Scott Krayenhoff, Ashley Broadbent, Matei Georgescu

**Affiliations:** † School of City & Regional Planning, Georgia Institute of Technology, Atlanta, Georgia 30332, United States; ‡ University of Michigan Institute for Social Research, Ann Arbor, Michigan 48106, United States; § University of Michigan School of Public Health, Ann Arbor, Michigan 48109, United States; ∥ School of Geographical Sciences and Urban Planning, Arizona State University, Tempe, Arizona 85281, United States; ⊥ School of Architecture, Georgia Institute of Technology, Atlanta, Georgia 30332 United States; # School of Electrical and Computing Engineering, Georgia Institute of Technology, Atlanta, Georgia 30332, United States; ∇ University of Texas Health Science Center at Houston School of Public Health, Austin, Texas 78701, United States; ○ Department of Health Sciences, Northeastern University, Boston, Massachusetts 02115, United States; ◆ School of Human Evolution and Social Change, Arizona State University, Tempe, Arizona 85281, United States; ¶ Taubman College of Architecture and Urban Planning, University of Michigan, Ann Arbor, Michigan 48109, United States; †† School of Environmental Sciences, University of Guelph, Guelph N1G2W1, Canada

**Keywords:** extreme heat event, infrastructure failure, heat-related mortality, climate change, urban heat management, compound climat, infrastructure failure events

## Abstract

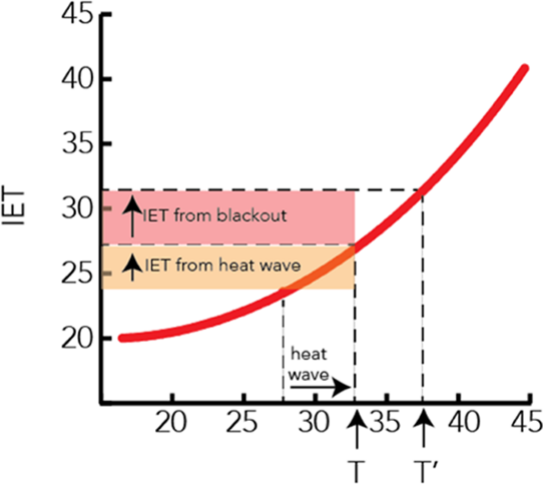

The recent concurrence of electrical grid failure events in time with extreme temperatures is compounding the population health risks of extreme weather episodes. Here, we combine simulated heat exposure data during historical heat wave events in three large U.S. cities to assess the degree to which heat-related mortality and morbidity change in response to a concurrent electrical grid failure event. We develop a novel approach to estimating individually experienced temperature to approximate how personal-level heat exposure changes on an hourly basis, accounting for both outdoor and building-interior exposures. We find the concurrence of a multiday blackout event with heat wave conditions to more than double the estimated rate of heat-related mortality across all three cities, and to require medical attention for between 3% (Atlanta) and more than 50% (Phoenix) of the total urban population in present and future time periods. Our results highlight the need for enhanced electrical grid resilience and support a more spatially expansive use of tree canopy and high albedo roofing materials to lessen heat exposures during compound climate and infrastructure failure events.

## Abstract 2

This study estimates the potential heat-related health impacts of concurrent heat wave and blackout events across three large U.S. cities.

## Introduction

**Figure 1:**
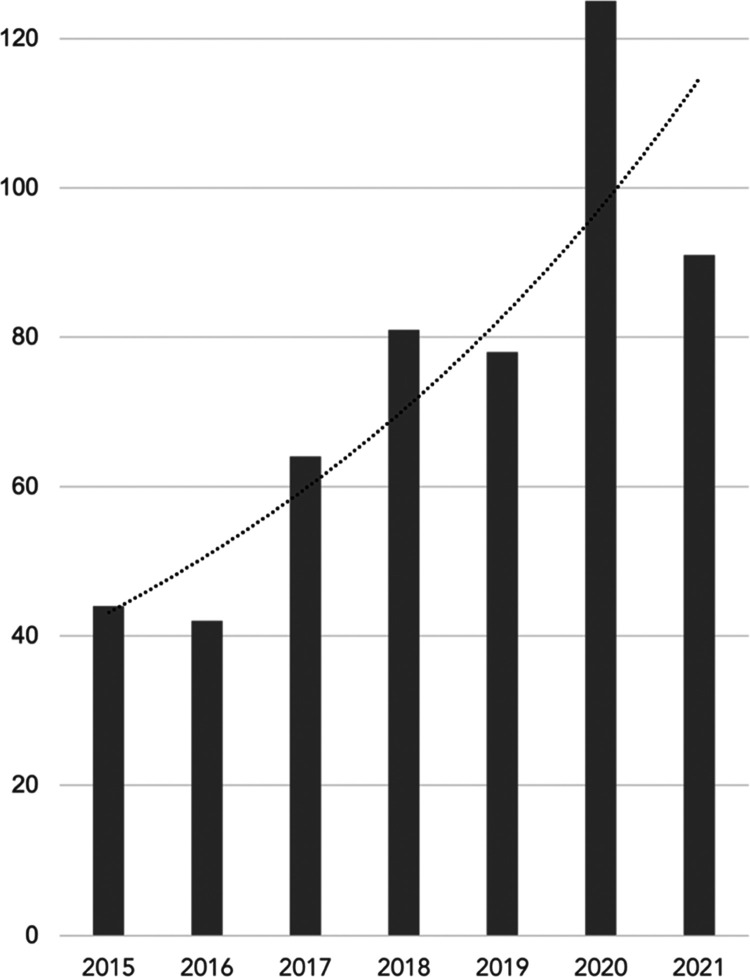
Total number of major electrical grid failure events for U.S. power utilities (2015–2021).^6^

The incidence of electrical grid failure or “blackout” events is increasing across the United States. Since 2015, when the U.S. Energy Information Administration commenced monthly reporting on major blackout events (defined as power outages lasting more than 1 h and impacting more than 50,000 customers), the number of such events nationwide has more than doubled, increasing by 151% between 2015–16 and 2020–21 (Figure 1). The majority of these events occurred during the summer months, when the annual demand for electricity is maximized and electrical grids are further stressed by extreme weather in the form of heat wave, hurricane, tornado, and wildfire events. Prior work finds the concurrence in time of two or more extreme weather events, such as a hurricane and heat wave, also to be on the rise,^1^ further enhancing potential health impacts of compound climate and infrastructure failure events. Most recently, in June of 2021, electrical grid failures associated with a heat wave of historical intensity in the Pacific Northwest of the United States resulted in a loss of power to tens of thousands of customers, at least 600 excess deaths, and more than 3500 emergency department visits for heat illness.^2−4^ Persistent drought conditions in the Western United States are further reducing the capacity of regional electrical generation and distribution systems to manage surges in electrical demand in this and other regions confronting climate-driven pressures on critical infrastructure.^5^

Despite a rising incidence of electrical grid failures, few studies have sought to directly assess the public health risks of compound climate and infrastructure failure events. Here, we estimate heat-related mortality and morbidity resulting from heat wave events of historical duration and intensity occurring simultaneously with simulated blackout conditions for the full urban populations of Atlanta, Georgia; Detroit, Michigan; and Phoenix, Arizona. Building on prior work modeling building-interior heat exposures resulting from concurrent heat wave and blackout events across these three cities,^7,8^ in this paper, we estimate the likelihood of health impacts through the development of an individual-level heat risk model accounting for hourly indoor and outdoor heat exposures and person-level risk characteristics. We then simulate the influence of urban heat management strategies on heat-related mortality and morbidity, as well as the impact of more intense heat wave events projected with continued global scale warming in future time periods.

This paper reports results from the Three-City Heat and Electrical Grid Failure Adaptation Study (3HEAT), which is focused on compound climate and infrastructure failure events in three large U.S. cities characterized by distinct regional climates and population characteristics. The cities of Atlanta, Georgia; Detroit, Michigan; and Phoenix, Arizona were selected to include three distinct climate zones in which approx. 75% of the U.S. population resides, as well as variable demographic and climatic risk factors for heat illness. Table 1 presents key characteristics for each city of relevance to heat vulnerability. We limit our study to three cities due to the extensive data inputs required to model both outdoor and building-interior heat exposures for more than 2.5 million residents across the three cities.

**Table 1:** Key Charateristics for Each City of Relevance to Heat Vulnerability

attribute	Atlanta	Detroit	Phoenix
population size^9^	420,000	713,770	1,445,630
male population^9^	49%	48%	51%
population > 65 years of age^9^	10%	12%	9%
nonwhite population^9^	62%	89%	34%
persons in poverty^9^	26%	38%	23%
central air conditioning in home	94%	53%	99%
mean summer daily temperature range^10^	21–32 °C (71–89 °F)	17–28 °C (63–82°F)	28–41 °C (82–105°F)
climate zone^11^	mixed-humid	cold	hot–dry

A collaboration between Arizona State University, the Georgia Institute of Technology, and the University of Michigan, the 3HEAT study examines three questions largely unaddressed by the literature on climate and health: (1) How does individual heat exposure change during concurrent heat wave and blackout events, relative to heat wave conditions with a fully operational electrical grid? (2) How do estimated public health impacts, including heat-related mortality and morbidity, change during concurrent heat wave and blackout events, relative to heat wave conditions with a fully operational electrical grid? (3) How do these estimated health impacts change in response to citywide urban heat management planning and to a growing intensity of heat wave conditions over time?

The first of these three study questions requires that we model heat exposure for both outdoor and building-interior environments. Established approaches to estimating health impacts associated with heat exposure are typically reflective of outdoor temperatures only, often measured at a single location, such as a metropolitan airport weather station, and therefore do not account for variable exposures for individuals in different locations of the city.^12^ Given that a grid failure has only limited impacts on outdoor temperatures but may substantially elevate building-interior temperatures, a reliance on outdoor temperature alone also fails to capture the changing nature of heat exposure during concurrent heat wave and blackout events.

To address this limitation of conventional approaches to assessing heat risk, we derive for this study a heat exposure metric referred to in prior work as “individually experienced temperature” or IET.^13,14^ IET provides a metric of human heat exposure that accounts for exposure changes as individuals move between indoor and outdoor environments during a 24 h period. Under blackout conditions, indoor heat exposures will rise, particularly for individuals who would otherwise have access to mechanical air conditioning in the home when the electrical grid is operational. The result is a rise in their IET and subsequent heat risk. We report the magnitude of this exposure change for different building types in a series of prior papers.^7,8,15^

To assess how elevated heat exposures during concurrent heat wave and blackout conditions may impact health outcomes, we devise a novel approach to estimating individual heat risk. Established exposure-response functions widely used in heat risk assessment are responsive to outdoor temperature measurements only, often derived from the same single location for a full urban population.^16−19^ Using urban-scale climate models, in combination with building energy simulation tools, we spatially disaggregate heat exposure to the level of the residential parcel, accounting for both how ambient temperatures vary across the urban environment and how these environmental temperatures influence building-interior temperatures for different classes of residential structures.

**Figure 2:**
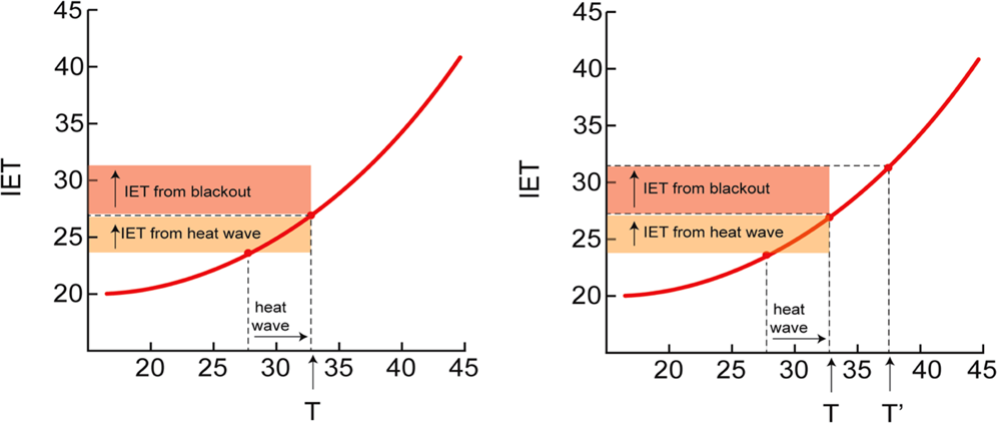
Estimation of “analog” temperature (*T*′). Weather station temperature (*T*) underestimates individually experienced temperature (IET) during blackout conditions (left panel). *T*′ adjusts *T* to capture both outdoor and building-interior heat burden reflected in IET (right panel). The red curve depicts the statistical association between IET and *T* under heat wave conditions with an operational grid, measured in celsius.

Health impact exposure-response functions for IET—reflective of both outdoor and building-interior temperatures—are not currently available. Therefore, we derive an “analog” outdoor temperature (*T*′) for each resident through the statistical association between estimated building-interior temperatures and a single airport weather station temperature (*T*) for every hour of the day during heat wave conditions with an operational electrical grid (the normal operating conditions captured by published exposure-response functions). Based on this statistical association, we then adjust outdoor temperatures to scale with rising IET (due to building-interior temperatures) during blackout conditions, yielding an analog outdoor temperature that can be used with established exposure-response functions for heat-related mortality and morbidity. The result is an outdoor temperature metric (*T*′) adjusted to reflect an elevated indoor heat burden during blackout conditions (Figure 2).

We address the last of these three questions—How do health impacts change in response to urban heat management strategies and future period warming?—through the re-simulation of IET in response to a modified built environment (in the form of enhanced tree canopy and high albedo roofing materials). The modified built environment has the general effect of lowering the analog air temperatures used to estimate individual heat risk. However, more intense heat waves in future time periods result in elevated analog temperatures, serving to enhance heat risk.

## Materials and Methods

Our research accomplished two principal tasks: (1) estimation of individually experienced temperature (IET) and (2) estimation of population health impacts from a concurrent heat wave and blackout event. Each of these tasks is described in turn.

### Individually Experienced Temperature (IET)

Estimation of IET required three sets of data inputs: (1) modeled outdoor ambient temperatures, (2) modeled building-interior temperatures, and (3) daily activity patterns for each resident, allowing for the individual assignment of location-specific outdoor and building-interior temperatures by time of day. We simulated outdoor and building-interior temperatures during historical heat wave events in each city with the Weather Research and Forecasting (WRF) model and the U.S. Department of Energy EnergyPlus building energy model (version 8.6), respectively. Historical heat wave events were classified as any 5 day period between 1980 and 2009 in which daily average air temperatures met or exceeded the 97.5th percentile of daily average temperature. Subsequently, the 90th percentile heat wave from each 30 year period in each city was selected and considered representative of the base period climate. The resulting heat wave periods identified were August 14–18, 1995 (Atlanta); June 15–19, 1994 (Detroit); and July 20–24, 2006 (Phoenix). Projected future heat waves representative of ∼2055 (mid-century) and ∼2085 (late century) were identified in the same way (i.e., 90th percentile 5 day heat waves in the global climate model output^20^) and subsequently simulated with WRF.

To capture the dynamics of an urban setting, the building effect parameterization (BEP) multilayer urban canopy model was selected in WRF. High-resolution (1 km^2^ grid spacing for the inner domain) two-way coupled WRF + BEP simulations were carried out using a nested grid configuration, enabling the downscaling of the large-scale synoptic flow from the coarsest outer grids to the finest inner grids across each of the three study regions. We made use of RCP 8.5 for both mid- and late-century time periods to assess the highest emission scenarios and to incorporate continuing uncertainty over achievable global emission reductions this century. Trees are represented in WRF-BEP as a simplification of the BEP-Tree model.^21^ Tree shading of impervious surfaces (streets and building walls) is calculated according to Beer’s law, accounting for forward transmission of shortwave radiation through leaves. For simplicity and to conserve energy, all shortwave radiation intercepted by tree foliage is assumed to be converted to transpiration and therefore does not impact air temperature via sensible heat flux. This represents a maximum impact scenario of well-watered trees on air temperature. In the current formulation, trees do not impact wind or longwave radiation, both factors that can slightly counter the shading and transpiration cooling effect. A more complete description of the tree implementation is given in Stone et al.^7^

To simulate building-interior exposures, we adapted the EnergyPlus model to simulate indoor temperatures within distinct residential building types, including 1-story, single-family houses; 2-story, single-family houses; and multistory apartment buildings. Building prototype parameters, including age, size, construction materials, and insulation values, were selected to reflect the local residential building stock in Atlanta, Detroit, and Phoenix. In addition to building prototype characteristics, the building energy model simulations were driven by the ambient weather output at the neighborhood (1 km) scale derived from the WRF model runs in each city. The location-specific effects of tree shading and shading from adjacent buildings were not captured in our modeling. For a complete description of our approach to building-interior temperature simulation, please see Stone et al.^7^

Individual hourly exposures to outdoor and building-interior temperatures were constructed from a synthetic population dataset for each city and national time–activity pattern survey data. The unavailability of location-specific identifiers for individuals in U.S. Census data requires that “synthetic” population datasets be developed for a range of demographic and economic analyses linking individual residents to geographic boundaries more disaggregated than Census blocks. For this study, we acquired synthetic population data from RTI International (RTI Synthpop), which depicts each resident of a Census block group as a randomly positioned point within the block group boundaries and assigns to these points demographic characteristics including age, sex, race, and income. As the total number of residents per block group is reported by the U.S. Census Bureau, but not the specific address of each resident, each person included in the RTI Synthpop digital map must be assigned to a specific address, enabling a set of building characteristics to be associated with each resident.

To carry out this task, we made use of a geographic information system (ESRI ArcGIS) to assign each resident to a unique address and residential structure through the creation of a circular buffer around each multifamily building or single-family house. First, any synthetic persons located within a 500-foot radius of a high-rise multifamily building (defined as more than five stories in height) were assigned to that building. Second, any synthetic persons located within a 250-foot radius of a low-rise multifamily building (defined as five stories in height or less) were assigned to that building. Lastly, all remaining synthetic persons were assigned to the nearest single-family home. This procedure resulted in the assignment of specific residential structure characteristics, including building-interior temperatures, to each resident of the three cities.

Individual time–activity patterns were derived from the American Time Use Survey (ATUS), conducted annually by the U.S. Bureau of Labor.^22^ We extracted time use data from respondents in each of the three study cities during the period 2004–2015. There were 2958 respondents that met the inclusion criteria and completed a total of 59,774 logged activities on the survey. Each activity was assigned to one of five location categories based on the nature of the recorded activity code, using guidance from Hoehne et al.^23^ The location categories include indoor home, indoor away (e.g., at a workplace), outdoor home, outdoor away, and vehicle. The time–activity profiles of ATUS respondents from each respective city were then assigned to each synthetic resident based on common attributes between the two datasets, including age, sex, occupation, and income. In assigning time–activity profiles to synthetic residents of each city, we drew randomly from the ATUS respondents who were assigned to the same combination of age, sex, occupation, and income categories. IETs for each hour were calculated for each synthetic person as the proportion of time spent in each of the five environmental conditions (indoor home, indoor away, outdoor home, outdoor away, vehicle) multiplied by the temperature in each of the respective environment and then summed for each hour of the day.

Building-interior temperatures were simulated in response to ambient heat wave conditions for two sets of electrical grid scenarios for the same 5 day heat wave period: (1) the grid is assumed to be fully operational (Power On) and (2) the grid is assumed to be fully nonoperational (Power Off) across the municipal extent of each city. For the Power On scenario, we made use of a mechanical air conditioning prevalence model to estimate the probability that central air conditioning, partial air conditioning (window AC units), or no mechanical air conditioning is available within each residential structure. Requiring as input parcel tax records reporting the home value, owner-occupied status, housing age, and housing structure type, combined with data on cooling-degree days within each city, AC prevalence was estimated with a regression model found in prior work to predict the presence of central or partial AC systems at the parcel level with a model accuracy of 84 and 82%, respectively.^24^ Building-interior temperatures during the Power On scenario are responsive to the type (or absence) of air conditioning system assigned to each structure. For structures with central AC systems, we assumed a constant set-point temperature of 24 °C during Power On conditions.^7^

For all scenarios in which the electrical grid is nonoperational, we assume 48 h of complete blackout conditions for every residential structure across the three cities and then restore power over the remaining 72 h of the heat wave based on the proximity of each structure to electrical grid substations. Electrical power is assumed to be restored to residential structures most proximate to these substation nodes first, with structures more distant from these nodes assigned a lower temporal priority in power restoration.^25^ In prior work, we found the median duration of recent U.S. blackout events impacting 1 million customers or more to be approx. 120 h, consistent with the large-scale events simulated herein.^8^ During blackout conditions, building-interior temperatures are responsive to localized outdoor temperatures and the specific characteristics of the building prototype (e.g., single vs multistory). For all residential structure types, windows are assumed to be opened at any time during concurrent heat wave and blackout conditions that ambient ventilation would have the effect of lowering indoor temperatures. Under the Power Off scenario, all residents of each city were assumed to remain in their residential structures (the indoor home location) due to the assumed inoperability of transportation systems and blackout conditions at the workplace.

IET is estimated for each synthetic resident based on daily time–activity patterns, simulated outdoor and building-interior temperatures associated with each activity, and outdoor and building-interior temperatures associated with variable heat exposure scenarios. Each exposure scenario is detailed in Table 2. We report ambient heat wave temperatures and mean IET under the Power On and Power Off scenarios by city in Table 3 and illustrate the distributions of these scenarios in Supporting Information Figure S1.

**Table 2:** Exposure Scenarios

scenario name	time period	electrical grid status	scenario conditions
power on	present day	operational	thermostat set point of 24 °C (75 °F) for residential structures with central AC systems.
power off	present day	nonoperational	no mechanical cooling for any structures for 48 h; power restoration sequenced by proximity to electrical substations over 72 h.
street trees	present day	nonoperational	all roadways with exception of interstate highways assumed to be 50% covered by tree canopy (tree characteristics are broadly representative of each region).
cool roofs	present day	nonoperational	roof material albedos for all buildings set at 0.88.
mid century	2050s	nonoperational	outdoor temperatures representative of a modeled heat wave in ∼2055 in each city for the RCP 8.5 scenario; no heat management strategies in effect.
late century	2080s	nonoperational	outdoor temperatures representative of a modeled heat wave in ∼2085 in each city for the RCP 8.5 scenario; no heat management strategies in effect.

**Table 3:** Ambient Heat Wave Temperatures and Mean IET under the Power On and Power Off Scenarios by City

attribute	Atlanta	Detroit	Phoenix
heat wave temperature range	25–36 °C (77–97 °F)	22–35 °C (72–95 °F)	32–45 °C (90–113 °F)
mean IET for Power On scenario	25.9 °C (78.7 °F)	25.3 °C (77.5 °F)	26.3 °C (79.3 °F)
mean IET for Power Off scenario	28.1 °C (82.5 °F)	26.9 °C (80.4 °F)	32.7 °C (90.8 °F)
mean IET for Street Tree scenario	27.6 °C (81.7 °F)	26.5 °C (79.7 °F)	32 °C (89.6 °F)
mean IET for Cool Roof scenario	27.5 °C (81.5 °F)	26.3 °C (79.3 °F)	31.4 °C (88.6 °F)

### Health Impact Assessment

We estimated the probability of heat-related mortality or morbidity for each resident during the 5 day heat wave and in response to each of the scenario conditions presented in Table 2. The health impact assessment consists of the following steps:

Step 1: Hourly outdoor temperatures from the nearest airport weather station in each city were downloaded from the Integrated Surface Database^26^ for a 10 day period, including five consecutive days of historic heat wave conditions and five subsequent days not characterized by heat wave conditions. We make use of airport weather station data to match the meteorological data used in the derivation of RR for emergency department visits in Atlanta and Phoenix and most commonly used to derive RR for heat-associated mortality.

Step 2: For both the extreme heat and nonextreme heat days, average daily IETs were joined with average daily airport temperatures for each resident by day (i.e., each day’s average outdoor temperature was associated with an individual’s estimated average IET for the same day).

Step 3. We then used the derived IET-outdoor temperature associations for unique combinations of building type, age, sex, income, and occupation to estimate “analog” outdoor temperature (*T*′) or the inflated temperature corresponding to blackout IET values, for these building type and demographic combinations. To do so, a mixed effect linear regression was fit to model IET as a function of outdoor airport temperature and building characteristics, age, sex, income, and occupation as follows
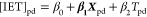
where p indexes persons, d indexes days, and ***X*** is a vector of individual characteristics (age, income, sex, occupation categories, housing categories).

The regression equation was rearranged to estimate an analog daily *T*, or *T*′, for each person’s daily IET
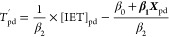
In this resulting equation, *T*′ for each person for a given IET is the estimated rate at which *T* increases with each degree of IET, or , and  is a constant, or intercept, specific to that type of person.

Step 4. We next substituted *T*′ for airport temperature in exposure-response functions to capture the effects of blackout conditions on individual heat risk. Gasparrini et al.^18^ derived nonlinear exposure-response functions for heat-related mortality for a large number of global cities, including Atlanta, Detroit, and Phoenix. Exposure-response functions for heat-related emergency department (ED) visits are only available for two of our three study cities: Atlanta^27^ and Phoenix.^17^ Details on each of these exposure-response functions are provided in Table 4.

**Table 4:** Details on Exposure-Response Functions

study	city and time period	health outcome	age and sex	threshold high T (°C)	RR for high vs threshold T	ln(RR) for 1 °C increase
Gasparrini et al. 2015^18^	Atlanta, 1985–2006	nonexternal all-cause mortality	all	25.6–32.5	1.064	0.0129
Gasparrini et al. 2015^18^	Detroit, 1985–2006	nonexternal all-cause mortality	all	23.9–31.3	1.58	0.110
Gasparrini et al. 2015^18^	Phoenix, 1985–2006	nonexternal all-cause mortality	all	33.3–40.6	1.21	0.0539
Winquist et al. 2016^27^	Atlanta, 1993–2012	heat-related ED visits	all	27–32	4.59	0.305
Petitti et al. 2016^17^	Phoenix, 2000–2011	heat-related ED visits	all	33–40	3.50	0.179

Step 5. The total daily burdens were calculated as the product of the age/sex/race category population and the category-specific incidence rate obtained for each health outcome: mean daily all-natural-cause mortality^28^ and cause-specific ED visits.^29^ The attributable fractions, derived from the nonextreme heat relative risks (RRs) for each person-day, were then multiplied by the total daily burdens. This estimated the fractional burdens for each person-day on a nonextreme heat day.

Step 6. For the extreme heat days in each city, the heat-attributable burdens were subtracted from the total daily burden to derive the “baseline health burden” or non-heat-associated burden for each age/sex/race category.

Step 7. The extreme heat days in our study were unusually high-temperature events and may therefore have resulted in mortality and morbidity beyond the annual rates observed in the total health burden data. Therefore, rather than using the attributable fractions from step 5, which assume that the attributable burdens were fractions of observed total burdens, we multiplied the extreme-heat-event RRs by the baseline burdens from step 6 to estimate the extreme-heat-event-attributable burdens for each person-day. Thus, the baseline burdens from step 6 allow estimated attributable burden to exceed the observed historical total burden (please see Supporting Information Figure S2). As a final step, we sum the heat-attributable burdens over the 5 day heat wave to estimate the total risk of heat-related mortality or morbidity. Estimated aggregate risks are limited to a maximum of 1 per person.

## Results and Discussion

**Figure 3:**
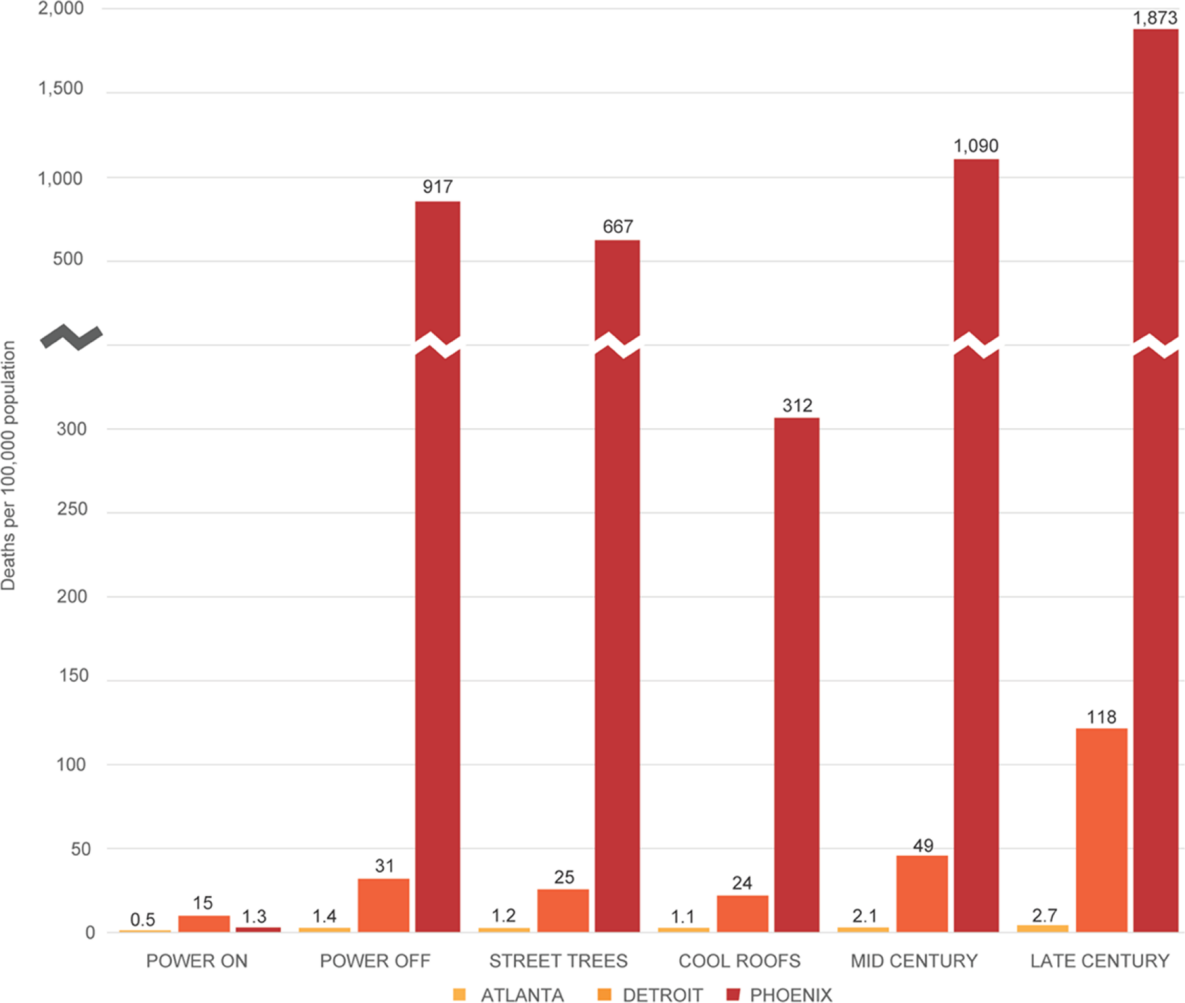
Rate of heat mortality during concurrent 5 day heat wave and blackout events in Atlanta, Detroit, and Phoenix by scenario. The Street Trees, Cool Roofs, Mid Century, and Late Century scenarios reflect blackout conditions.

We find a substantial increase in heat-related mortality and morbidity across each of the three cities with a loss of electrical power during heat wave conditions. As presented in Figure 3, the rate of mortality (per 100,000 population) under conditions of an operational electrical grid (Power On) shows substantial variation between Atlanta and Phoenix, cities with a citywide prevalence of central AC in residential structures of greater than 90%, and Detroit, which exhibits a population rate of AC prevalence of less than 60%.^8^ During a heat wave event of historical intensity with an operational electrical grid, Detroit experiences a rate of heat-related mortality many-fold greater than the cities with high AC prevalence due to a lower adaptive capacity for heat in the form of mechanical cooling.

The estimated rate of heat-related mortality (per 100,000 population) for each city during simulated heat wave conditions with an operational electrical grid (Power On scenario) is 0.5 in Atlanta (total = 2), 15 in Detroit (total = 107), and 1.3 in Phoenix (total = 19) (Note: Total heat-related deaths are not reported in Figure 3). In addition to AC prevalence, the relatively low level of mortality in Atlanta can be attributed to maximum heat wave temperatures 9 °C lower than observed in Phoenix, which reaches 45 °C in the late afternoon.

We find a citywide loss of electrical power during a 5 day heat wave of historical intensity (Power Off scenario) to more than double the estimated rate of heat mortality in Atlanta and Detroit, where maximum outdoor and building-interior temperatures reach about 35 °C in the late afternoon and remain above a daily minimum temperature of 25 °C (Atlanta) or 22 °C (Detroit). In Phoenix, where the lowest daily high temperature over the 5 day heat wave is 43 °C and daily minimum temperatures average 32 °C, the rate of heat-related mortality increases by about 700% relative to the Power On scenario, reflecting the extremity of heat exposures in a desert city in the absence of mechanical AC. As reported in Figure 3, the estimated rate of heat-related mortality for the Power Off scenario in Phoenix is 917 (approximate total =13,250 deaths), which approaches 1% of the synthetic population. The estimated rate of heat-related mortality for comparable 5 day events in Atlanta and Detroit is 1.4 (total = 6) and 31 (total = 221), respectively.

Heat management strategies in the form of street trees and cool roofing measurably reduce heat-related mortality across the three cities, particularly in Phoenix. An increase from present-day tree canopy shading of roadways to an average of 50% across all streets is found to reduce the estimated rate of heat mortality by 14 and 19% in Atlanta and Detroit, respectively, and by 27% in Phoenix. In response to the installation of highly reflective cool roofs for all building types citywide, the rate of heat-related mortality falls by 21% in Atlanta, 23% in Detroit, and 66% in Phoenix. We attribute the disproportionate benefits of cool roofing in Phoenix to the extremity of temperatures during heat wave conditions, during which rates of evapotranspiration from trees may be reduced, lessening the cooling effects of tree canopy.^30^

Projected warming by middle (∼2055) and late (∼2085) century under the RCP 8.5 global emissions trajectory further amplifies estimated heat-related mortality, particularly by late century. In response to a mid-century heat wave of comparable intensity to the historical event modeled in the base period, the rate of heat-related mortality increases by 50% in Atlanta, 58% in Detroit, and 19% in Phoenix relative to the base period. By late century, the rate of heat-related mortality increases by 93%, 281%, and 104% in Atlanta, Detroit, and Phoenix, respectively. Relative to population size, which remains fixed at present-year levels for the future warming scenarios, the rate of heat mortality in Phoenix from a concurrent heat wave and blackout event is projected to exceed 1% of the total modeled population in mid-century and approach 2% of the population by late century.

**Figure 4:**
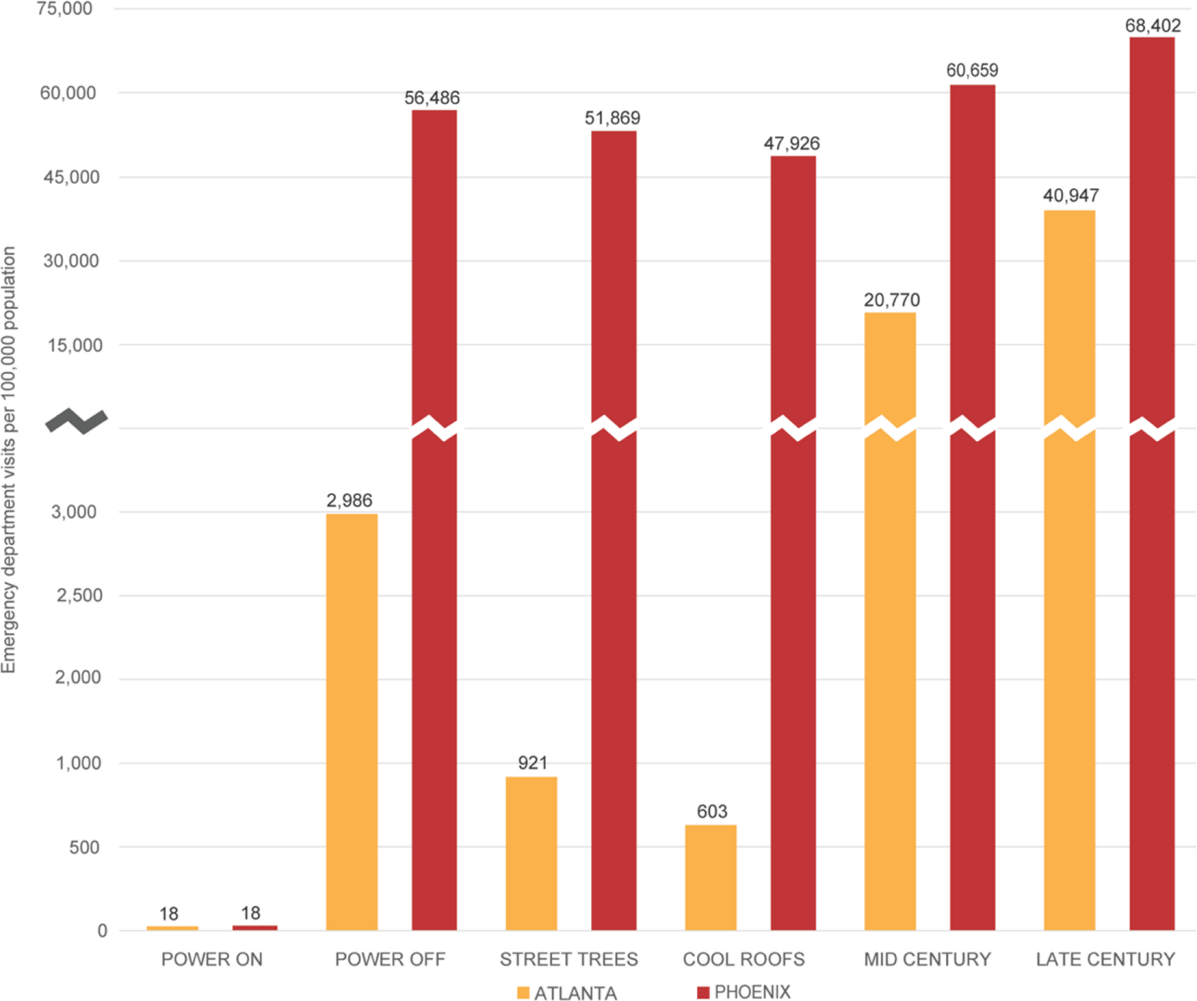
Emergency department visits per 100,000 population during concurrent 5 day heat wave and blackout events in Atlanta and Phoenix by scenario. The Street Trees, Cool Roofs, Mid Century, and Late Century scenarios reflect blackout conditions. No published exposure-response function for emergency department visits is available for Detroit.

The estimated number of ED visits resulting from a 5 day heat wave substantially increases in response to simulated blackout conditions. Based on health surveillance data recording daily emergency department visits for heat-related conditions, exposure-response functions have been derived to associate daily ED visits with temperature change in two of our three study cities: Atlanta and Phoenix.^17,27^ Employing the same analog temperature approach used to estimate heat-related mortality, we estimate the number of ED visits in response to our six scenarios in Figure 4. Under current conditions, a citywide blackout increases the rate of heat-related ED visits (per 100,000 population) in Atlanta from 18 (total = 76) to almost 3,000 (approximate total = 12,540) over the 5 day event, representing approx. 3% of the population. In Phoenix, the estimated rate of heat-related ED visits increases from 18 (total = 260) under the Power On scenario to more than 56,000 (approximate total = 816,570) visits under the Power Off scenario, representing more than 50% of the population.

Citywide urban heat management strategies reduce heat-related morbidity associated with a concurrent heat wave and blackout event in both cities. An expansion of street trees to 50% shading over roadways reduces the rate of ED visits by almost 70% in Atlanta and 8% in Phoenix. The conversion of all building roofs to cool materials reduces the rate of ED visits by 80% in Atlanta and 15% in Phoenix.

Accounting for more intense heat waves in future years, a concurrent heat wave and blackout event by mid-century increases the ED visit rate in Atlanta approx. 7-fold to more than 20,000 per 100,000 population. The ED visit rate in Phoenix increases by 7% over the base year Power Off scenario. By late century, the fraction of the urban population requiring emergency medical services from the simulated compound climate and infrastructure failure event is estimated as more than 40% in Atlanta and 68% in Phoenix.

## Discussion

We estimate a magnitude of health risks associated with a compound climate and infrastructure failure event across the three large U.S. cities that is well in excess of prior work focused on extreme heat events in urbanized areas.^31,32^ Our measure of IET, accounting for individual-level heat exposures responsive to localized outdoor and indoor climates, provides a more accurate metric of individual heat exposure than the use of single, stationary outdoor temperature sensors at regional weather stations. While the substitution of IET in exposure-response functions does not result in elevated estimates of heat wave mortality or morbidity relative to prior studies when electrical grids are assumed to be operational,^33,34^ the simulated loss of mechanical AC for residents with access to AC systems greatly elevates indoor heat exposures, serving in turn to elevate the analog temperatures used herein to estimate health impacts. Simulated blackout conditions for a historical 5 day heat wave more than doubled the rate of heat mortality in Atlanta and Detroit and increased the rate of heat mortality in Phoenix—where average heat wave temperatures exceed 37 °C—to almost 1% of the total urban population.

The more pronounced risk of heat mortality and morbidity in Phoenix relative to Atlanta and Detroit can be attributed both to the higher temperatures experienced during an extreme heat wave event and a disproportionate impact of blackout conditions for cities with a high AC prevalence. Ambient heat wave temperatures in Phoenix reach a maximum intensity 9–10 °C greater than daily high temperatures in Atlanta or Detroit, while minimum temperatures only fall to 32 °C, sustaining high heat exposures over a full 24 h period for successive days. In addition to more intense heat wave conditions for residents of Phoenix, the loss of electrical power produces a disproportionately greater shift in individual heat exposures than estimated for residents of Atlanta or Detroit. Reported in Table 3, the shift in IET resulting from a loss of electrical power during heat wave conditions is 2.2 and 1.6 °C in Atlanta and Detroit, respectively, and more than 6 °C in Phoenix. Phoenix is not only hotter than Atlanta or Detroit, the shift in individual heat exposures in response to a loss of AC is about 3-fold greater, on average, than for residents of Atlanta or Detroit—an outcome that results from both high heat wave temperatures and an AC prevalence rate that approaches 100%. Protective of health during heat wave periods with an operational electrical grid, high AC prevalence may have the unintended effect of amplifying heat vulnerability during grid failure events.

While none of the cities included in the study has experienced a concurrent heat wave and blackout event of the intensity and duration modeled herein, recent surveillance data focused on the unhoused population in Phoenix find a heat-mortality rate comparable to that estimated to result from a citywide compound climate and infrastructure failure event. The Maricopa County Public Health Agency, which serves more than 90% of the Phoenix metropolitan population, reported 130 heat-related deaths among an unhoused population of approx. 8200 during 2021 or a heat-mortality rate of 1580 per 100,000.^35,36^ Representing approx. 1.6% of the estimated total unhoused population, this estimate of the heat-mortality rate among a population lacking regular access to shelter or mechanical air conditioning falls within the same order of magnitude of what we estimate to result from a concurrent heat wave and blackout event for the full Phoenix population (∼1%). An important limitation of this reference dataset for the Phoenix population, however, is the surveillance period, which is reflective of a full warm season as opposed to a single, intense heat wave event. In addition, differences in the baseline health and acclimatization to extreme heat between these two populations may yield different outcomes.

Our estimates do not account for individuals’ behavioral adaptations or the actions of first responders during such a severe heat crisis. In reality, if a blackout event was regionally localized, many individuals would be able to leave their homes and travel to cooler locations. We also do not account for potential governmental responses to a concurrent heat wave and blackout, such as evacuation of the most vulnerable to heat illness or the deployment of mobile power generators for cooling centers. Other important sources of uncertainty in our analysis include (1) the degree to which measures of relative risk based on outdoor temperatures are reflective of indoor heat exposures, (2) uncertainties in the precision of the ambient and indoor temperature models used for the study, (3) uncertainties in behaviors and locations of individuals throughout the day and hence their daily IETs, and (4) uncertainties in the exposure-response functions in the applied epidemiology studies (including the changing nature of population susceptibility with a period of prolonged heat exposure).

These limiting assumptions notwithstanding our results suggest that the extent of heat morbidity in Atlanta and Phoenix resulting from a concurrent heat wave and blackout event carries the potential to overwhelm regional emergency medical systems. With almost three percent of the urban population in Atlanta estimated to require emergency medical care and more than half of the Phoenix population, the capacity of regional emergency departments to effectively treat heat illness would be exceeded in both cities (Atlanta has less than 2000 emergency department beds;^37^ Phoenix has less than 3000 emergency department beds^38^). Importantly, the exposure-response functions used in this study reflect heat morbidity rates during historical surveillance periods in which regional electrical grids are fully or largely operational. The inability of regional emergency medical systems to treat widespread heat illness during periods of electrical system inoperability due to the large number of residents requiring medical care may indicate that a higher rate of heat mortality than estimated by our approach would result.

The substantial magnitude of heat risk in Phoenix, with more than 50% of the population at risk of heat illness from blackout conditions during a heat wave of historical intensity, suggests the imperative for a high level of electrical grid resilience and back-up power generation, particularly for critical facilities such as hospitals. Burillo et al.^39^ found the potential for cascading electrical grid failures across Arizona to increase 30-fold in response to a 1 °C rise in average annual temperatures, suggesting a growing potential for compound events over time. Most critical is investments in back-up power generation at regional cooling centers to ensure critical public health protections during blackout conditions and an expansion in the number of cooling centers to accommodate larger populations during compound climate and infrastructure events. We estimate that the total number of cooling centers in each of the three study cities would likely accommodate less than 1–2% of the urban population in the event of a concurrent heat wave and blackout event.^8^ As the frequency and intensity of extreme heat events continues to rise, all cities should be working to enhance electrical grid resilience during heat wave conditions.

The extremity of the estimated risk for heat-related mortality in Phoenix and of heat morbidity in both Atlanta and Phoenix, highlight the need for more comprehensive emergency response preparations by local, state, and federal officials. Such plans should address emergency cooling needs, the provision of drinking water should water treatment and delivery systems fail, and the potential need for evacuation and temporary housing for residents lacking access to personal vehicles. At present, state-level hazard mitigation plans in Arizona, Georgia, and Michigan do not identify an electrical grid failure during an extreme heat event as a defined class of hazard. The Arizona Department of Health Services identifies an electrical grid failure event during the summer months as requiring the highest level of governmental emergency response but offers limited details on what responses would be deployed.^40^ In light of the rising frequency of electrical grid failure events, in concert with a rising incidence of extreme weather events nationwide, compound climate and infrastructure failure events should be directly addressed through governmental hazard mitigation and emergency response planning.

Conventional urban heat management strategies, including an enhancement of citywide street tree canopy and the use of high albedo roofing materials, potentially carry significant benefits for lessening heat risk during concurrent heat wave and blackout events. Across the three study cities, street trees lessened heat-related mortality by an average of 20%, while cool roofing could lessen heat-related mortality by an average of 37%. Both strategies also were highly effective at reducing estimated heat morbidity in Atlanta and Phoenix, with the modeled increase in street trees and cool roofing associated with an average reduction in ED visits of 39 and 48%, respectively. While we did not simulate the effects of both enhanced street tree area and cool roofing simultaneously, other studies have found the combination of these strategies to yield a marginally lower cooling effect than the sum of these two strategies modeled independently.^41,42^ Importantly, our approach estimates benefits of cool roofing for both reduced ambient and building-interior temperatures, while street trees are not assumed to shade buildings in our modeling, and thus, no building-interior reductions in temperature from direct tree shading were considered. Prior work accounting directly for the benefits of tree shading for indoor climates finds significant cooling benefits that are not reflected in our results.^43^ Moreover, our estimate of reduced mortality from expanded tree canopy does not account for direct radiative effects on (i.e., direct shading of) pedestrians and thus may underestimate the full benefits of this strategy.

We assume an extent of tree canopy shading over roadways that exceeds present canopy extents in each of the study cities but likely would be achievable over time for all but the largest roadways. While citywide data on street tree coverage over roadways are not available for Atlanta, Detroit, or Phoenix, data for Chicago, as an example, find streets to be shaded by an average of 35% when measured at the Census block group level, with 14% of all block groups found presently to have tree shading of roadways at 50% or greater,^44^ illustrating the potential to achieve this level of canopy cover over streets in a highly urbanized environment. Likewise, we set roof albedo values at levels presently achievable with commercially available products for both commercial and residential roofs. We believe the heat management targets set in this study are ambitious but physically attainable over time in each of the study cities, albeit with differing levels of investment needed in supporting infrastructure and more pronounced constraints on water resources for irrigation in Phoenix.

A rising potential for compound climate and infrastructure failure events in large cities of the U.S. highlights a pronounced vulnerability of urban populations confronting more extreme heat in all regions of the country.^45^ In this study, we find the health impact of a multiday electrical grid failure event during heat wave conditions, and in the absence of cooling interventions, to be substantially greater than the estimated levels of heat mortality and morbidity associated with a heat wave alone. We further find this amplifying effect of infrastructure failure on health outcomes to rise rapidly by middle to late century, as heat wave intensities exceed historical levels. Widespread physical changes to the built environment of cities, enhancing evapotranspiration, shading, and solar reflection, carry the potential to measurably reduce estimated levels of heat illness and death.

## Supporting Information Available

Distribution of IET by city for the Power On, Power Off, Cool Roof, and Street Tree scenarios in the present time period (Figure S1) and extrapolated RR curves based on ref 18 for Atlanta, Detroit, and Phoenix (Figure S2) (PDF)

## Notes

Study datasets are available through Dryad: https://doi.org/10.5061/dryad.cz8w9gj67.
