# Supplementary material for: How Blackouts during Heat Waves Amplify Mortality and Morbidity Risk
Source: Environ Sci Technol. 2023 May 23;57(22):8245–55. doi: 10.1021/acs.est.2c09588 (PMC10249403; doi:10.1021/acs.est.2c09588)
Supplement: Supplementary file 1 — es2c09588_si_001.pdf [file es2c09588_si_001.pdf]

**How Blackouts during Heat Waves Amplify Mortality and Morbidity Risk**

Authors: Brian Stone Jr. [1*], Carina J. Gronlund[2,3], Evan Mallen[1], David Hondula[4], Marie S. O'Neill[3], Mayuri Rajput[5], Santiago Grijalva[6], Kevin Lanza[7], Sharon Harlan[8,9], Larissa Larsen[10], Godfried Augenbroe[5+], E. Scott Krayenhoff[11], Ashley Broadbent[4], Matei Georgescu[4]

[1]School of City & Regional Planning, Georgia Institute of Technology, Atlanta, Georgia 30332, United States of America

[2]University of Michigan Institute for Social Research, Ann Arbor, Michigan 48106, United States of America

[3]University of Michigan School of Public Health, Ann Arbor, Michigan 48109, United States of America

[4]School of Geographical Sciences and Urban Planning, Arizona State University, Tempe, Arizona 85281, United States of America

[5]School of Architecture, Georgia Institute of Technology, Atlanta, Georgia 30332 United States of America

[6]School of Electrical and Computing Engineering, Georgia Institute of Technology, Atlanta, Georgia 30332, United States of America

[7]University of Texas Health Science Center at Houston School of Public Health, Austin, Texas 78701, United States of America

[8]Department of Health Sciences, Northeastern University, Boston, Massachusetts 02115, United States of America

[9]School of Human Evolution and Social Change, Arizona State University, Tempe, Arizona 85281, United States of America

[10]Taubman College of Architecture and Urban Planning, University of Michigan, Ann Arbor, Michigan 48109, United States of America

[11]School of Environmental Sciences, University of Guelph, Guelph N1G2W1, Canada

* Corresponding author
Email: stone@gatech.edu
[+] Deceased (May 14, 2021)

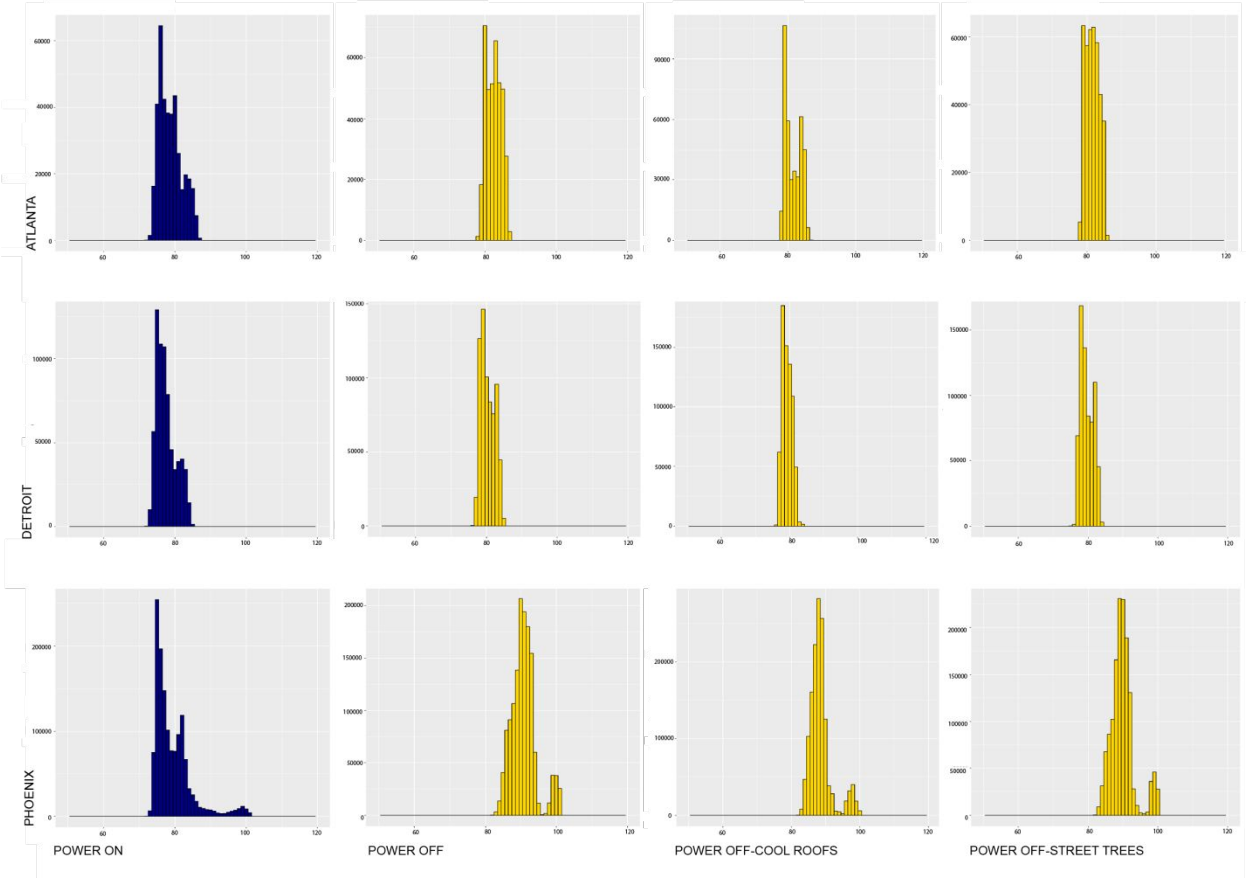


**Figure S1**: Distribution of IET by city for the Power On, Power Off, Cool Roof, and Street Tree scenarios in the present time period.

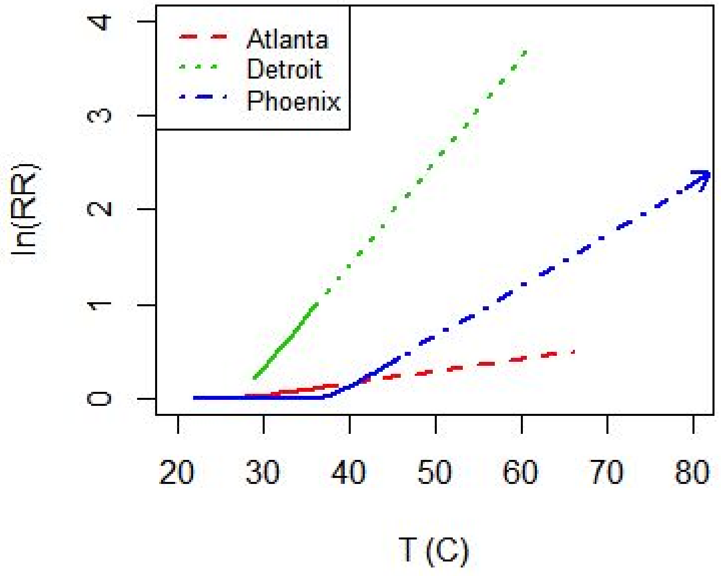


**Figure S2**: Extrapolated RR curves based on [18] for Atlanta, Detroit, and Phoenix.
